# The Role of Diet in Tinnitus Onset: A Hospital-Based Case-Control Study from Italy

**DOI:** 10.3390/nu15030621

**Published:** 2023-01-25

**Authors:** Carlotta Micaela Jarach, Alessandra Lugo, Werner Garavello, Piet A. van den Brandt, Anna Odone, Christopher R. Cederroth, Cristina Bosetti, Silvano Gallus

**Affiliations:** 1 Department of Environmental Health Sciences, Istituto di Ricerche Farmacologiche Mario Negri IRCCS, 20156 Milan, Italy; 2 Department of Otorhinolaryngology, School of Medicine and Surgery, University of Milan-Bicocca, 20121 Milan, Italy; 3 GROW—School for Oncology and Developmental Biology, Department of Epidemiology, Maastricht University Medical Centre, 6211 Maastricht, The Netherlands; 4 CAPHRI—School for Public Health and Primary Care, Department of Epidemiology, Maastricht University Medical Centre, 6211 Maastricht, The Netherlands; 5 School of Medicine, University Vita-Salute San Raffaele, 20132 Milan, Italy; 6 Department of Public Health, Experimental and Forensic Medicine, University of Pavia, 27100 Pavia, Italy; 7 Laboratory of Experimental Audiology, Department of Physiology and Pharmacology, Karolinska Institutet, 171 77 Stockholm, Sweden; 8 Nottingham Biomedical Research Centre, National Institute for Health Research (NIHR), Nottingham University Hospitals NHS Trust, Ropewalk House, Nottingham NG1 5DU, UK; 9 Division of Clinical Neuroscience, Hearing Sciences, School of Medicine, University of Nottingham, Nottingham NG7 2UH, UK; 10 Department of Oncology, Istituto di Ricerche Farmacologiche Mario Negri IRCCS, 20156 Milan, Italy

**Keywords:** tinnitus, case-control study, risk factors, epidemiology, diet, lifestyles

## Abstract

Knowledge on the role of diet in tinnitus onset is mostly based on few cross-sectional studies. In 2016–2019 we conducted a hospital-based case-control study in northern Italy on 185 incident idiopathic tinnitus cases and 198 controls, providing data on dietary habits through a 37-item food-frequency questionnaire. Odds ratios (OR) for tinnitus risk were derived through unconditional multiple logistic regression models. Moderate-to-high vs. low intake of caffeine (OR, 0.49; 95% confidence interval (CI), 0.24–0.99) and butter (OR, 0.46; 95% CI, 0.23–0.93), and high vs. low intake of poultry (OR, 0.43; 95% CI, 0.23–0.81), prosciutto (OR, 0.44; 95% CI, 0.23–0.85), and legumes (OR, 0.50; 95% CI, 0.28–0.92) were inversely associated with tinnitus onset. Other food items, including cereals, red meat, fish, vegetables, and fruit did not show any statistically significant relationship. The variety of food consumed decreased the risk of tinnitus (OR for at least 20 vs. less than 16 different food items, 0.47; 95% CI, 0.24–0.90). Our findings highlight the importance of diet in tinnitus onset and confirm a potential inverse association of protein-rich food and caffeine on the incidence of tinnitus. Confirmation of our findings in longitudinal studies is necessary before proving any diet recommendations for tinnitus prevention.

## 1. Introduction

Tinnitus is a common disorder defined as the perception of noise, typically a buzzing, rustling, or whistling sound, in the absence of an external acoustic stimulus [1]. Despite its high prevalence and debilitating consequences [2], the available epidemiological data on its risk factors are inadequate. Above all, there is a distinct paucity of large, well-conducted analytical studies, i.e., case-control or cohort studies, that quantify the association between putative risk factors and the onset of tinnitus [2,3]. Currently, tinnitus management often involves education and counselling, with a general recommendation of a healthy diet; modifiable lifestyle risk factors for tinnitus, of which diet is an example, should be identified and addressed to prevent and manage tinnitus symptoms [4]. In a recent systematic review and meta-analysis of tinnitus risk factors, Biswas and colleagues [3] revealed the scant evidence on exposures causally related to tinnitus. The review found hearing loss, temporo-mandibular joint disorder, depression, chronic obstructive pulmonary disease, and hyperlipidaemia to be tinnitus risk factors. Most of this evidence comes from cross-sectional studies, i.e., study designs unable to infer causality; of 374 articles analysed, only 49 were case-control or cohort studies [3]. Among those analytical studies, besides a few investigations on alcohol drinking or obesity, only the Nurses’ Health Study II evaluated diet (specifically caffeine intake) as a potential risk factor for tinnitus [5]. In this large US female cohort, higher caffeine intake was associated with a lower risk of incident tinnitus [5].

To our knowledge, only two other articles explored the relationship between diet and tinnitus onset using longitudinal data. Tang and colleagues, in the US Blue Mountains Hearing Study, showed that lower intake of fruit fibre and cereal fibre were significantly associated with a 55–65% increased risk of developing tinnitus over 10 years [6], and that intake of dietary flavonoids did not seem to protect against tinnitus incidence [4]. 

Gallus et al. performed a survey in 2015, which reported the latest prevalence of tinnitus in Italy [7]. Tinnitus was found in 6.2% of the participants, men and women having similar prevalence rates (6.0% and 6.4%, respectively). Severe tinnitus afflicted 1.2% of the population, and prevalence increased with increasing age. Diet, specifically the Mediterranean one, could have a role in prevention of tinnitus onset and partly explain the relatively limited prevalence of tinnitus in areas of the Mediterranean basin [7]. This is confirmed by various researchers who postulated a potential protective influence of certain foods on tinnitus onset or annoyance [8,9,10,11,12], either Mediterranean food items (fresh fruit and vegetable intakes [10]) or other nutrients (reduced intake of vitamin B_2_ and B_3_ [8], chocolate intake [9], and improved intake of vitamin D [11] and manganese [12]), leading to preventive recommendations and, in some circumstances, even treatment recommendations, through dietary supplements. However, to date, there is no conclusive evidence to support the latter recommendations. 

In the absence of either a treatment that can definitively cure tinnitus or instruments that can manage the symptomatology in all patients, preventing tinnitus onset plays an even more fundamental role. For this reason, we designed a case-control study aiming to shed light on potential dietary influencing factors for this debilitating neurological disorder.

## 2. Materials and Methods

In 2016, the Mario Negri Institute in Milan (Italy), in collaboration with the San Gerardo Hospital (Monza e Brianza, Italy), began a case-control study on incident cases to investigate various possible determinants of tinnitus. Our data were collected between June 2016 and May 2019. The study protocol was approved by the Ethics Committee of the Local Health Unit of Monza Brianza (Comitato Etico Brianza, 26 January 2016). All individuals gave their written informed consent to participate in the study.

Cases were outpatients admitted to the ear, nose, and throat (ENT) department without a previous diagnosis of tinnitus, but identified by an ENT surgeon as idiopathic tinnitus. Anamnesis was collected, and patients underwent a complete ENT medical examination to exclude ear canal or ear drum pathologies, as well as cerumen impaction, otitis, tympanic membrane perforations, middle and inner ear pathologies, otosclerosis, acoustic neuromas, and vestibular pathologies. Cases reporting psychiatric or neurological comorbidities were also excluded, due to the interference of these conditions with tinnitus perception, as it has been demonstrated that a comorbid psychological disorder might influence the perceived severity of tinnitus and quality of life [13]. Controls were patients without tinnitus enrolled in surgery departments of San Gerardo Hospital, in agreement with heads of the corresponding departments. They were admitted to the ENT department for either non-otologic problems (46%), orthopaedic disorders (18%), surgical conditions (20%), or miscellaneous other illnesses, including eye (9%), skin (<1%), pneumological (<1%), and urologic disorders (<2%).

Originally, frequency-matching (age and sex) was planned, but not reached, as the recruitment stopped for the COVID-19 restrictions. Overall, 383 individuals aged 18 years or older were enrolled (185 cases and 198 controls), and undertook an audiogram and impedance audiometry. 

### 2.1. Audiogram and Impedance Audiometry

Both cases and controls had audiograms and impedance audiometry conducted to determine their acoustic threshold, which could prevent them from being included in the study. Cases underwent a Magnetic Resonance Imaging (MRI) of the cerebellopontine angle in order to detect acoustic neurinoma: 4 patients were excluded from the study because of this reason. Pure tone audiometry was performed in a sound-proof booth and was used to detect individuals’ acoustic threshold—the smallest intensity of a sound that a person needs to detect its presence. Air-conduction and bone-conduction were detected: for air conduction, signals were transmitted through headphones, while a vibratory stimulus applied to mastoid process was used for bone-conduction. Bone conduction was tested masking contralateral air-conduction. According to the primary audiogram, patients with conductive or mixed hearing loss were excluded. The hearing threshold was defined as the mean hearing threshold of both right and left ears on pure-tone audiometry tests at 500, 1000, 2000, and 4000 Hz. Participants with <25 dB were defined as normal hearing individuals, while those with ≥25 dB as individuals with hearing loss (mild or severe).

### 2.2. Questionnaire

Trained interviewers collected data on both cases and controls through a face-to-face survey, using a structured CAPI (Computer Assisted Personal Interview) questionnaire. Information collected included sociodemographic characteristics (e.g., sex, age, level of education, marital status), anthropometric factors, tobacco smoking (e.g., never, current, or former smokers), alcohol consumption (e.g., no use, less than 7 glasses per week, 7 or more glasses per week), lifestyle habits (e.g., physical activity), medical history (including anxiety or depressive symptoms, sleep difficulties, hyperacusis, exposure to noises, snoring, or obstructive sleep apnoea syndrome), and medications. For cases only, questions regarding tinnitus included its duration, lateralization, and severity (assessed using the Italian validated version of the Tinnitus Handicap Inventory, THI Cronbach alpha 0.9 [14]). Concerning dietary habits, diet was checked through a 37-item food frequency questionnaire (FFQ) which was slightly modified from one used in early case-control studies on selected digestive tract cancer sites; this FFQ was previously validated in the 90s [15,16]. Number of portions per week (each portion weight based on the usual Italian diet) were collected for cereals (pasta and rice, bread, whole wheat bread, potatoes, pastries, and sugar), protein-rich foods (red meat, poultry, legumes, fish, eggs, sausages, liver, ham, and prosciutto), fat-rich foods (milk, cheese, olive oil, seed oil, margarine, and butter), vegetables (carrots, spinach, tomatoes, peppers, salad, cabbages, and total vegetables), and fruit (apples and pears, oranges, melon, and total fruit). For condiments, consumption was measured asking for the subjective perception of use, as no use, scarce, normal or high. Drinking habits (coffee, decaffeinated coffee, tea, cola, and water consumption) were also assessed. Caffeine intake was computed considering the consumption of either coffee, tea, or cola drinks. To derive the caffeine intake, we used the BDA database, an Italian food composition database [17]. For each dietary item, participants were asked about their habits during the previous year, before the symptoms which led them to the hospital appeared. In order to study food diversity, we created a variable counting how many of the 28 solid foods (excluding oil, butter, margarine, and drinks) were regularly consumed (at least one portion per week). The variable therefore theoretically ranged from 0 to 28.

### 2.3. Statistical Analysis

To analyse the relationship between dietary habits and tinnitus, we computed odds ratios (ORs) and 95% confidence intervals (CIs) for tinnitus onset through unconditional multiple logistic regression models, after adjustment for sex, age, level of education, body mass index (BMI), smoking status, alcohol consumption, and hearing loss. When computing ORs, we categorized food items into approximately tertiles/quartiles in order to create homogeneous groups. χ^2^ tests were used to test trends in categorical variables. The software SAS 9.4 (Cary, North Carolina, USA) was used for statistical analyses. All data were managed anonymously.

## 3. Results

Our sample was composed of 185 cases (56.2% men and 43.8% women) and 198 controls (51.0% men, 49.0% women). Cases were, on average, three years older than controls, but this difference was not significant (55.8 years, standard deviation (SD) 16.2 for cases; 52.8 years, SD 16.2, for controls; *p* value 0.074). No statistically significant relationship was found among cases and controls according to level of education, BMI, alcohol consumption, or hearing loss, while ex-smokers had significantly less frequent tinnitus compared to never smokers (see Supplementary Materials, Table S1). In all, 35.3% of tinnitus cases had hearing loss versus 21.8% of the controls.

Table 1 shows the ORs for tinnitus onset according to selected dietary items found to be significantly associated. We found an inverse association for moderate—but not for high—intake of caffeine, between 850 and 1749 mg per week (compared to those reporting a low or null caffeine intake, the OR for those reporting a high intake of caffeine was 0.49; 95% CI, 0.24–0.99; *p* for trend 0.277). Three or more portions (one portion defined as 150 g) of poultry per week were protective for tinnitus onset (compared to those reporting zero or one portion per week, the OR for those reporting three or more portions of poultry per week was 0.43; 95% CI, 0.23–0.81; *p* for trend 0.009). At least two portions per week of prosciutto (one portion defined as 50 g) and legumes (one portion defined as 100 g) were protective in tinnitus onset (compared to those reporting occasional or no consumption of prosciutto, the OR for those reporting at least two portions per week was 0.44; 95% CI, 0.23–0.85; *p* for trend 0.019; compared to those reporting occasional or no consumption of legumes, the OR for those reporting at least two portions per week was 0.50; 95% CI, 0.28–0.92; *p* for trend 0.023). Those who reported a normal or high use of butter had a significantly reduced risk of tinnitus onset (compared to those who do not use butter, the OR for those reporting normal or high use of butter was 0.46; 95% CI, 0.23–0.93; *p* for trend 0.055). One portion of pasta per day (80 gr), compared to maximum three portions per week, was protective on tinnitus onset, although not statistically significant (OR 0.62; 95% CI, 0.34–1.13), as well as eating more than one portion of bread per day (with one portion defined as 50 gr) compared to no consumption (OR 0.59; 95% CI, 0.25–1.39). No statistically significant associations have been observed with other dietary item including meat, fish, cheese, fruit and vegetables and drinks, including tea, cola, milk and water (see Supplementary Materials, Table S2). 

In Table 1, as well as in Supplementary Tables S1 and S2, ORs and corresponding 95% CIs for cases with tinnitus vs. controls are also shown strati-fied by sex. Legumes resulted to be protective on tinnitus onset in men only: compared to those reporting occasional or no consumption of legumes, the OR in men for those reporting at least two portions per week was 0.21; 95% CI, 0.08–0.54, *p* for trend 0.001. Similarly, when stratified, inverse association for moderate - but not for high - use in-take of caffeine was found in men only: compared to those reporting a low or null use of caffeine intake, the OR for those reporting high intake of caffeine was 0.33, 95% CI, 0.11–0.99, *p* for trend 0.597). 

In men, bread portions resulted protective on tinnitus onset: compared to those reporting no intake of bread, the OR in those reporting 1-5 portions per week was 0.17 (95% CI, 0.05–0.57) and in those reporting eight or more portions per week it was 0.21 (95% CI, 0.06–0.71, *p* for trend 0.063).

Poultry and prosciutto resulted to be protective on tinnitus onset in women only: compared to those reporting zero or one portion per week, the OR for those reporting three or more portions of poultry per week in women was 0.38; 95% CI, 0.15–0.94, *p* for trend 0.042. In women, compared to those reporting occasional or no consumption of prosciutto, the OR for those reporting at least two portions per week was 0.27; 95% CI, 0.09–0.77, *p* for trend 0.011.

In terms of food diversity, the risk of tinnitus onset decreased with increasing number of foods (including sugar) regularly consumed at a weekly basis (compared to individuals reporting less than 16 different items, the OR for a weekly meal composed of at least 20 different items was 0.47; 95% CI, 0.24–0.90; *p* for trend 0.022). This pattern was observed in women, but not in men: compared to individuals reporting the consumption of less than 16 different items, ORs were 0.27 (95% CI, 0.10–0.73) for those reporting the consumption of 16-19 different food items and 0.38 (95% CI, 0.14–0.98) for those reporting at least 20 different items p for trend 0.031). Food diversity has not shown to be statistically significant for tinnitus onset in men.

## 4. Discussion

To the best of our knowledge, this is the first analytical study that extensively assesses the variety of dietary items as predictors of tinnitus: a diversified diet seems to be protective against tinnitus onset.

The scientific literature on the relationship between food and tinnitus is not particularly robust, nor wide. In a 2019 review, Hofmeister [18] attempted to assess the efficacy of diet and supplements in the management of the condition, while also drawing attention to the paucity of rigorous analytical studies on the matter. Several of their results are consistent with our findings: in particular, the review cites a study which indicated that tinnitus-related annoyance was linked to poor protein consumption [8], and a study showing that a high intake of proteins was associated with reduced odds of tinnitus [19]. We confirmed these findings, having found that higher consumption of protein-rich foods, such as poultry and legumes, significantly decrease the risk of tinnitus onset.

A varied diet has been shown to have favourable implications for human health [20]. Our results confirm those who have previously emphasized the importance of nutrition quality and variety. Spankovich et al. [21] found that individuals who reported better food quality (measured using the Healthy Eating Index, HEI) had lower odds of reporting persistent tinnitus. They also found that participants with better dietary variety were less likely to report persistent tinnitus (OR, 0.64; 95% CI, 0.43–0.94) [21].

Given the potential interactions between caffeine and the central nervous system [18], some researchers have suggested reducing coffee consumption to alleviate, at least, tinnitus-related discomfort, although there is a dearth of data indicating the usefulness of such a waiver [18]. In contrast, two cross-sectional studies [22,23] and one prospective cohort study [5] consistently found that higher caffeine intake was associated with a lower prevalence or incidence of tinnitus. We recognize that reverse causality is plausible, at least in the cross-sectional studies, as tinnitus sufferers frequently complain of sleeplessness, which is undoubtedly exacerbated by coffee drinking. Our results from incident cases add further evidence which supports the hypothesis of a protective function of caffeine. However, a randomized control trial found that 300 mg of caffeine did not significantly alter tinnitus discomfort [24].

Few studies have found a favourable effect of fruit and vegetables intake on tinnitus incidence or frequency [6,10]; however, our study was unable to confirm these associations.

The differences in risk found between sexes appeared interesting and deserve further investigation using prospective studies.

The analytical design of our study is among its main strengths; conducting a case-control study allows the analysis of numerous risk variables concurrently, and is useful for drawing an initial evaluation of potential associations. Identifying these potential risk factors by valid and well-designed studies leads to more robust hypotheses for tinnitus prevention, as well as for possible tinnitus treatments. Moreover, another important strength is that we used only items from an FFQ whose reproducibility was assessed in the literature, specifically in hospital case-control designs [15]. Additionally, we enrolled patients with incident tinnitus only. Ideally, case-control studies use incident cases to avoid the difficulty of separating factors related to causation and survival, although studies have often been conducted using prevalence data [25].

A key disadvantage of our case-control design, on the other hand, is recall bias: we attempted to avoid it by inquiring about the participants’ dietary habits in the year preceding the beginning of the symptoms, and designing a hospital-based study instead of a population-based one, where controls would have been healthier. Secondly, from a statistical perspective, we were unable to adjust for energy intake, as our questionnaire did not allow us to derive it. However, when further adjusted for number of portions, which could be seen as a proxy of energy intake, results did not change substantially. At the same level, our questionnaire was not designed to measure dietary patters or to derive a Mediterranean diet score. This is unfortunate, as some research has speculated a possible favourable role of the Mediterranean diet as a partial preventive factor for tinnitus [7]. A third limitation is that with the items at our disposal, we were not able to reproduce any validated dietary diversity score as proposed in the literature; moreover, for condiments, we did not provide a definition of how many grams were “scarce”, “normal”, or “high”.

A further limitation is the sample size; patients’ enrolment (foreseen 600 cases and 600 controls) was early stopped because of the COVID-19 pandemic, thus impeding us from obtaining an adequate statistical power. Furthermore, the earlier stop in enrolment did not allow us to obtain data to categorize food items based on the Food and Agriculture Organization of the United Nations (FAO) guidelines or other nutritional recommendations, but only a categorization based on our tertiles/quartiles. Frequency-matching was initially planned, but we were unable to conclude the recruitment because of pandemic restrictions. Nevertheless, it is important to highlight that our design, even with a modest sample size, included incident cases only, reducing the risk of information bias compared to prevalent cases; however, information bias cannot be excluded. This characteristic, in our opinion, further elevates our results, giving them considerable relevance for the broadening of knowledge on the influencing factors of the onset of tinnitus. If our findings on food variety are confirmed by prospective studies, recommending a varied diet would be a strategic key-message for tinnitus prevention.

## 5. Conclusions

Our findings contribute to a better understanding of tinnitus and provide insights into potential preventative measures for this challenging condition. They highlight the importance of diet in tinnitus prevention, and confirm a potential protective effect of protein-rich foods and caffeine on the incidence of tinnitus. We found that moderate caffeine intake was inversely associated with tinnitus, but not higher caffeine intake. However, it is too early to derive causal interpretations and provide preventative recommendations based on the food–tinnitus inverse associations identified. Further research is needed to confirm and extend these findings and to determine the underlying mechanisms involved.

## Figures and Tables

**Table 1:** Distribution of 185 cases with incident diagnosis of tinnitus and 198 controls, overall and stratified by sex, according to selected dietary habits. Odds ratios (OR) and corresponding 95% confidence intervals (CI) for cases with tinnitus vs. controls. Italy, 2016–2019.

Dietary Item	Total			Men			Women		
	Cases N ° (%)	Controls N ° (%)	OR * (95% CI)	Cases N ° (%)	Controls N ° (%)	OR * (95% CI)	Cases N ° (%)	Controls N ° (%)	OR * (95% CI)
Caffeine mg/week									
<850 mg	70 (45.5)	56 (33.0)	1.00 ^∧^	32 (37.7)	24 (28.9)	1.00 ^∧^	38 (55.1)	32 (36.8)	1.00 ^∧^
850–1749 mg	40 (26.0)	52 (30.6)	**0.49 (0.24–0.99)**	21 (24.7)	25 (30.1)	**0.33 (0.11–0.99)**	19 (27.5)	27 (31.0)	0.58 (0.22–1.54)
≥1750 mg	44 (28.5)	62 (36.4)	0.69 (0.34–1.43)	32 (37.7)	34 (41.0)	0.75 (0.25–2.27)	12 (17.4)	28 (32.2)	0.55 (0.19-1.60)
*p* for trend			0.277			0.597			0.236
Poultry portions (each 150 g)/week									
0–1	73 (38.9)	66 (33.3)	1.00 ^∧^	36 (34.6)	29 (28.7)	1.00 ^∧^	37 (46.8)	37 (38.1)	1.00 ^∧^
2	53 (29.0)	47 (23.2)	1.13 (0.57–2.23)	31 (29.8)	28 (27.7)	1.05 (0.38–2.92)	22 (27.9)	19 (19.6)	1.15 (0.42–3.11)
≥3	57 (31.1)	85 (49.5)	**0.43 (0.23–0.81)**	37 (35.6)	44 (43.6)	0.48 (0.18–1.28)	20 (25.3)	41 (42.3)	**0.38 (0.15–0.94)**
*p* for trend			**0.009**			0.128			**0.042**
Prosciutto portions (each 50g)/week									
0–0.5	101 (55.8)	96 (48.5)	1.00 ^∧^	49 (47.1)	47 (46.5)	1.00 ^∧^	52 (67.5)	49 (50.5)	1.00 ^∧^
1	46 (25.4)	43 (21.7)	0.85 (0.44–1.66)	32 (30.8)	23 (22.8)	1.66 (0.62–4.47)	14 (18.2)	20 (20.6)	0.49 (0.17–1.38)
≥2	34 (18.8)	59 (29.8)	**0.44 (0.23–0.85)**	23 (22.1)	31 (30.7)	0.55 (0.21–1.40)	11 (14.3)	28 (28.9)	**0.27 (0.09–0.77)**
*p* for trend			**0.019**			0.325			**0.011**
Legumes portions (each 100g)/week									
0–0.5	73 (40.1)	61 (30.8)	1.00 ^∧^	47 (45.2)	29 (28.7)	1.00 ^∧^	26 (33.3)	32 (33.0)	1.00 ^∧^
1	42 (23.1)	45 (22.7)	0.87 (0.44–1.73)	22 (21.2)	20 (19.8)	1.09 (0.37–3.21)	20 (25.6)	32 (33.0)	0.70 (0.25–1.97)
≥2	67 (36.8)	92 (46.5)	**0.50 (0.28–0.92)**	35 (33.7	52 (51.5)	**0.21 (0.08–0.54)**	32 (41.0)	40 (41.2)	0.69 (0.28–1.74)
*p* for trend			**0.023**			**0.001**			0.445
Butter ^†^									
Not used	88 (47.6)	78 (39.4)	1.00 ^∧^	48 (46.2)	38 (37.6)	1.00 ^∧^	40 (49.4)	40 (41.2)	1.00 ^∧^
Scarce	61 (33.0)	68 (34.3)	0.98 (0.54–1.77)	33 (31.7)	31 (30.7)	1.08 (0.46–2.55)	28 (34.6)	37 (38.1)	0.88 (0.33–2.33)
Normal use or high use	36 (19.4)	52 (26.3)	**0.46 (0.23–0.93)**	23 22.12	32 31.68	0.45 (0.16–1.26)	13 (16.1)	20 (20.6)	0.45 (0.16–1.28)
*p* for trend			0.055			0.194			0.157
Pasta portions (each 80 g)/week									
0–3	74 (40.2)	70 (35.4)	1.00 ^∧^	31 (30.1)	28 (27.7)	1.00 ^∧^	43 (53.1)	42 (43.3)	1.00 ^∧^
4–6	54 (29.4)	41 (20.7)	0.83 (0.43–1.63)	35 (34.0)	19 (18.8)	0.88 (0.30–2.54)	19 (23.5)	22 (22.7)	0.70 (0.26–1.85)
≥7	56 (30.4)	87 (43.9)	0.62 (0.34–1.13)	37 (35.9)	54 (53.5)	0.77 (0.31–1.90)	19 (23.5)	33 (34.0)	0.62 (0.25–1.56)
*p* for trend			0.118			0.569			0.298
Bread portions (each 50 g)/week									
0	45 (25.3)	38 (19.2)	1.00 ^∧^	27 (26.5)	16 (15.8)	1.00 ^∧^	18 (23.7)	22 (22.7)	1.00 ^∧^
1–5	57 (32.0)	72 (36.3)	0.53 (0.26–1.06)	28 (27.5)	36 (35.6)	**0.17 (0.05–0.57)**	29 (38.2)	36 (37.1)	1.18 (0.43–3.25)
6–7	46 (25.8)	57 (28.8)	0.53 (0.25–1.14)	26 (25.5)	29 (28.7)	0.32 (0.09–1.07)	20 (26.3)	28 (28.9)	0.81 (0.27–2.49)
≥8	30 (16.9)	31 (15.7)	0.59 (0.25–1.39)	21 (20.6)	20 (19.8)	**0.21 (0.06–0.71)**	9 (11.8)	11 (11.3)	2.81 (0.53–14.8)
*p* for trend			0.220			0.063			0.598
Meat portions (each 150 g)/week									
0–1	100 (54.7)	112 (56.6)	1.00 ^∧^	48 (46.2)	46 (45.5)	1.00 ^∧^	52 (65.8)	66 (68.0)	1.00 ^∧^
2	37 (20.2)	34 (17.2)	1.49 (0.75–2.94)	24 (23.1)	20 (19.8)	1.87 (0.68–5.11)	13 (16.5)	14 (14.4)	1.39 (0.48–4.04)
≥3	46 (25.1)	52 (26.2)	0.97 (0.51–1.85)	32 (30.8)	35 (34.7)	1.33 (0.56–3.17)	14 (17.7)	17 (17.5)	0.72 (0.23–2.27)
*p* for trend			0.889			0.484			0.778
Fish portions (each 150 g)/week									
0–0.5	50 (27.6)	54 (27.3)	1.00 ^∧^	24 (23.3)	27 (26.7)	1.00 ^∧^	26 (33.3)	27 (27.8)	1.00 ^∧^
1	53 (29.3)	46 (23.2)	1.19 (0.59–2.38)	31(30.1)	26 (25.7)	1.50 (0.54–4.22)	22 (28.2)	20 (20.6)	0.96 (0.32–2.83)
≥2	78 (43.1)	98 (49.5)	0.75 (0.41–1.40)	48 (46.6)	48 (47.5)	0.91 (0.36–2.33)	30 (38.5)	50 (51.6)	0.67 (0.26–1.70)
*p* for trend			0.315			0.703			0.382
Cheese portions (each 25 g)/week									
0–2	101 (55.2)	116 (58.6)	1.00 ^∧^	54 (51.9)	59 (58.4)	1.00 ^∧^	47 (59.5)	57 (58.8)	1.00 ^∧^
3	32 (17.5)	28 (14.1)	1.29 (0.63–2.67)	21 (20.2)	11 (10.9)	1.48 (0.48–4.59)	11 (13.9)	17 (17.5)	0.95 (0.33–2.73)
≥4	50 (27.3)	54 (27.3)	0.85 (0.46–1.58)	29 (27.9)	31 (30.7)	0.59 (0.23–1.51)	21 (26.6)	23 (23.7)	1.52 (0.58–4.03)
*p* for trend			0.711			0.326			0.451
Vegetables portions ^⌠^ (each 150 g)/week									
0–5	53 (29.1)	63 (31.8)	1.00	35 (33.7)	39 (38.6)	1.00 ^∧^	18 (23.1)	24 (24.7)	1.00 ^∧^
6–7	50 (27.5)	51 (26.8)	0.77 (0.38–1.56)	31 (29.8)	30 (29.7)	0.56 (0.21–1.50)	19 (24.4)	21 (21.7)	1.34 (0.45–4.03)
≥8	79 (43.4)	84 (42.4)	0.75 (0.40–1.41)	38 (36.5)	32 (31.7)	0.65 (0.25–1.68)	41 (52.6)	52 (53.6)	1.01 (0.38–2.68)
*p* for trend			0.390			0.396			0.965
Fruit portions (each 150 g)/week									
0–5	59 (32.4)	64 (32.3)	1.00 ^∧^	36 (35.0)	34 (33.7)	1.00 ^∧^	23 (29.1)	30 (30.9)	1.00 ^∧^
6–7	38 (20.9)	43 (21.7)	0.96 (0.47–1.97)	24 (23.3)	25 (24.8)	0.88 (0.33–2.39)	14 (17.7)	18 (18.6)	1.13 (0.35–3.65)
≥8	85 (46.7)	91 (46.0)	0.78 (0.43–1.44)	43 (41.8)	42 (41.6)	0.81 (0.34–1.92)	42 (53.2)	49 (50.5)	0.71 (0.27–1.83)
*p* for trend			0.423			0.624			0.435
Food diversity									
0–15	77 (41.6)	51 (23.8)	1.00 ^∧^	37 (35.6)	26 (25.7)	1.00 ^∧^	40 (49.38	25 (25.8)	1.00 ^∧^
16-19	62 (33.5)	71 (35.9)	0.53 (0.28–1.00)	41 (39.4)	36 (35.6)	0.73 (0.29–1.85)	21 (25.93	35 (36.1)	**0.27 (0.10–0.73)**
≥20	46 (24.9)	76 (38.4)	**0.47 (0.24–0.90**)	26 (25.0)	39 (38.6)	0.54 (0.19–1.52)	20 (24.69	37 (38.1)	**0.38 (0.14–0.98)**
*p* for trend			**0.022**			0.242			**0.031**

Significant estimates at a 0.05 level are in bold. * Estimated using unconditional multiple logistic regression models after adjustment for sex, age, education, body mass index, smoking status, alcohol consumption, and hearing loss. ^∧^ Reference category. ° The sum does not add up to the total number of individuals due to some missing values. ^⌠^ All vegetables excluding potatoes and legumes. ^†^ Butter was measured asking for the subjective perception of use, as no use, scarce, normal or high.

## Data Availability

The data presented in this study are available on request from the corresponding author. The data are not publicly available because the study is still ongoing.

## Supplementary Materials

The following supporting information can be downloaded at: https://www.mdpi.com/article/10.3390/nu15030621/s1: Table S1, Distribution of 185 cases with incident diagnosis of tinnitus and 198 controls, according to selected socio-demographic characteristics and lifestyle habits. Odds ratios (OR) and corresponding 95% confidence intervals (CI) for cases with tinnitus vs. controls. Italy, 2016–2019; Table S2, Distribution of 185 cases with incident diagnosis of tinnitus and 198 controls, according to selected dietary habits which resulted not significant. Odds ratios (OR) and corresponding 95% confidence intervals (CI) for cases with tinnitus vs. controls. Italy, 2016–2019.
