# Supplementary material for: The Role of Diet in Tinnitus Onset: A Hospital-Based Case-Control Study from Italy
Source: Nutrients. 2023 Jan 25;15(3):621. doi: 10.3390/nu15030621 (PMC9920666; doi:10.3390/nu15030621)
Supplement: Supplementary file 1 [file nutrients-15-00621-s001.zip › nutrients-2059081-supplementary.pdf]

Supplementary Table S1. Distribution of 185 cases with incident diagnosis of tinnitus and 198 controls, overall and stratified by sex, according to selected socio-demographic characteristics and lifestyle habits. Odds ratios (OR) and corresponding 95% confidence intervals (CI) for cases with tinnitus vs. controls. Italy, 2016-2019.

	Total			Men			Women		
	Cases N (%)	Controls N (%)	OR* (95% CI)	Cases N (%)	Controls N (%)	OR* (95% CI)	Cases N (%)	Controls N (%)	OR* (95% CI)
Total	185	198	-	104 (50.7)	101 (49.3)	-	81 (45.5)	97 (54.5)	-
Sex									
Men	104 (56.2)	101 (51.0)	1.00^	-	-	-	-	-	-
Women	81 (43.8)	97 (49.0)	0.85 (0.49-1.47)	-	-	-	-	-	-
Age group (years)									
<45	41 (22.1)	61 (30.8)	1.00^	23 (22.1)	34 (33.7)	1.00^	18 (22.2)	27 (27.8)	1.00^
45-54	46 (24.9)	42 (21.2)	1.42 (0.70-2.89)	28 (26.9)	20 (19.8)	2.07 (0.72-5.98)	18 (22.2)	22 (22.7)	0.94 (0.33-2.67)
55-64	31 (16.8)	43 (21.7)	0.87 (0.39-1.95)	20 (19.2)	19 (18.8)	1.02 (0.31-3.30)	11 (13.6)	24 (24.7)	0.71 (0.21-2.43)
65+	67 (36.2)	52 (26.3)	1.67 (0.69-4.04)	33 (31.7)	28 (27.7)	1.09 (0.29-4.05)	34 (42.0)	24 (24.7)	2.59 (0.66-10.1)
P for trend			0.454			0.893			0.327
Level of education°									
No/elementary/middle school	58 (31.7)	48 (24.5)	1.00^	32 (31.0)	24 (23.7)	1.00^	26 (32.5)	24 (25.2)	1.00^
High school	64 (35.0)	73 (37.2)	0.94 (0.47-1.90)	36 (35.0)	34 (33.7)	1.01 (0.36-2.83)	28 (35.0)	39 (41.1)	0.90 (0.31-2.63)
University	61 (33.3)	75 (38.3)	0.75 (0.36-1.57)	35 (34.0)	43 (42.6)	0.47 (0.15-1.47)	26 (32.5)	32 (33.7)	1.03 (0.33-3.23)
P for trend			0.309			0.142			0.931
BMI									
Underweight or Normal weight	99 (53.5)	113 (57.1)	1.00^	46 (44.2)	42 (41.6)	1.00^	53 (65.4)	71 (73.2)	1.00^
Overweight	62 (33.5)	59 (29.8)	1.48 (0.81-2.70)	41 (39.4)	41 (40.6)	1.53 (0.66-3.57)	21 (25.9)	18 (18.6)	1.39 (0.53-3.65)
Obese	24 (13.0)	26 (13.1)	0.94 (0.43-2.10)	17 (16.4)	18 (17.8)	1.08 (0.38-3.07)	7 (8.6)	8 (8.3)	0.59 (0.14-2.52)
P for trend			0.731			0.696			0.837
Smoking status									
Never smokers	101 (54.6)	96 (48.5)	1.00^	44 (42.3)	42 (41.6)	1.00^	57 (70.4)	54 (55.7)	1.00^
Ex-smokers	55 (29.7)	68 (34.3)	**0.48 (0.26-0.90)**	42 (40.4)	37 (36.6)	0.62 (0.25-1.56)	13 (16.1)	31 (32.0)	**0.33 (0.13-0.87)**
Current smokers	29 (15.7)	34 (17.2)	0.75 (0.37-1.54)	18 (17.3)	22 (21.8)	0.49 (0.17-1.38)	11 (13.6)	12 (12.4)	1.42 (0.46-4.34)
P for trend			0.156			0.154			0.663

Alcohol consumption/week									
None	73 (55.7)	62 (44.3)	1.00^	22 (35.5)	25 (37.3)	1.00^	51 (73.9)	37 (50.7)	1.00^
0-6 glass	39 (29.8)	48 (34.3)	0.75 (0.41-1.36)	23 (37.1)	25 (37.3)	1.16 (0.48-2.80)	16 (23.2)	23 (31.5)	0.53 (0.22-1.28)
7+	19 (14.5)	30 (21.4)	0.56 (0.27-1.14)	17 (27.4)	17 (25.4)	1.11 (0.44-2.80)	2 (2.9)	13 (17.8)	**0.12 (0.02-0.60)**
P for trend			0.096			0.798			**0.005**
Hearing Loss°									
No	119 (64.7)	151 (78.2)	1.00^	67 (64.4)	73 (74.5)	1.00^	52 (65.0)	78 (82.1)	1.00^
Yes	65 (35.3)	42 (21.8)	1.33 (0.67-2.67)	37 (35.6)	25 (25.5)	1.06 (0.40-2.86)	38 (35.0)	17 (17.9)	1.61 (0.52-5.01)

Significant estimates at a 0.05 level are in bold.

*Estimated using unconditional multiple logistic regression models after adjustment for sex, age, education, body mass index, smoking status, alcohol consumption, and hearing loss

^ Reference category.

°The sum does not add up to the total number of the subjects due to some missing values.

Supplementary Table S2. Distribution of 185 cases with incident diagnosis of tinnitus and 198 controls, overall and stratified by sex, according to selected dietary habits not included in the main text. Odds ratios (OR) and corresponding 95% confidence intervals (CI) for cases with tinnitus vs. controls. Italy, 2016-2019.

	Total			Men			Women		
Dietary item°	Cases N (%)	Controls N (%)	OR* (95% CI)	Cases N (%)	Controls N (%)	OR* (95% CI)	Cases N (%)	Controls N (%)	OR* (95% CI)
Coffee cups/week									
<7	61 (33.3)	55 (27.8)	1.00^	25 (24.3)	25 (24.8)	1.00^	36 4(5.0)	30 (30.9)	1.00^
7-14	80 (54.7)	75 (37.9)	0.74 (0.40-1.37)	48 (46.6)	38 (37.6)	0.80 (0.31-2.05)	32 (40.0)	37 (38.1)	0.61 (0.25-1.46)
15+	42 (23.0)	68 (34.3)	0.64 (0.31-1.32)	30 (29.1)	38 (37.6)	0.66 (0.22-1.98)	12 (15.0)	30 (30.9)	0.41 (0.14-1.23)
P for trend			0.213			0.459			0.097
Decaffeinated cups/week									
0	127 (77.0)	141 (80.6)	1.00^	75 (83.3)	71 (84.5)	1.00^	52 (69.3)	70 (76.9)	1.00^
Occasional+	38 (23.0)	34 (19.4)	1.64 (0.85-3.17)	15 (16.7)	13 (15.5)	1.40 (0.49-4.02)	23 (30.7)	21 (23.1)	2.46 (0.95-6.42)
P for trend									
Tea cups/week									
0	64 (35.8)	58 (30.1)	1.00^	40 (39.6)	33 (34.4)	1.00^	24 (30.8)	25 (25.8)	1.00^
Occasional-1	51 (28.5)	62 (32.1)	0.65 (0.33-1.29)	34 (33.7)	36 (37.5)	0.58 (0.22-1.50)	17 (21.8)	26 (26.8)	0.78 (0.25-2.43)
2+	64 (35.8)	73 (37.8)	0.74 (0.39-1.41)	27 (26.7)	27 (28.1)	0.51 (0.19-1.37)	37 (47.4)	46 (47.4)	1.06 (0.42-2.69)
P for trend			0.346			0.177			0.813
Cola cups/week									
0	99 (58.2)	115 (62.9)	1.00^	51 (52.6)	52 (56.5)	1.00^	48 (65.8)	63 (69.2)	1.00^
Occasional-1	55 (32.4)	44 (24.0)	1.74 (0.92-3.29)	33 (34.0)	27 (29.4)	1.48 (0.58-3.75)	22 (30.1)	17 (18.7)	1.82 (0.68-4.91)
2+	16 (9.4)	24 (13.1)	0.68 (0.26-1.79)	13 (13.4)	13 (14.1)	0.67 (0.17-2.65)	3 (4.1)	11 (12.1)	0.65 (0.13-3.25)
P for trend			0.884			0.869			0.804
Whole wheat bread portions (each 50 g)/week									
0	66 (37.3)	73 (36.9)	1.00^	37 (37.4)	39 (38.6)	1.00^	29 (37.2)	34 (35.0)	1.00^
0.5+	111 (63.7)	125 (63.1)	1.11 (0.64-1.93)	62 (62.6)	62 (61.4)	1.03 (0.47-2.25)	49 (62.8)	63 (65.0)	1.12 (0.47-2.70)
Pastry portions (6 cookies each)/week									

0-2	60 (33.2)	75 (37.9)	1.00^	27 (26.2)	35 (34.7)	1.00^	33 (42.3)	40 (41.2)	1.00^
3-6	21 (11.6)	29 (14.6)	0.77 (0.34-1.71)	9 (8.7)	18 (17.8)	0.77 (0.23-2.53)	12 (15.4)	11 (11.3)	0.99 (0.29-3.40)
7+	100 (55.2)	94 (47.5)	0.90 (0.51-1.60)	67 (65.1)	48 (47.5)	1.57 (0.66-3.73)	33 (42.3)	46 (47.4)	0.42 (0.17-1.05)
P for trend			0.737			0.266			0.062
Sugar portions (each one spoon)/week									
0	77 (43.3)	75 (38.5)	1.00^	34 (34.0)	36 (36.0)	1.00^	43 (55.1)	39 (41.1)	1.00^
Occasional-7	54 (30.3)	54 (27.7)	0.93 (0.49-1.75)	33 (33.0)	25 (25.0)	1.40 (0.53-3.69)	21 (26.9)	29 (30.5)	0.62 (0.24-1.59)
8+	47 (26.4)	66 (33.8)	0.81 (0.43-1.53)	33 (33.0)	39 (39.0)	1.23 (0.50-3.06)	14 (18.0)	27 (28.4)	0.50 (0.18-1.39)
P for trend			0.528			0.638			0.158
Liver portions (each 150 g)/week									
0	141 (82.0)	161 (81.3)	1.00^	76 (77.5)	76 (75.3)	1.00^	65 (87.8)	85 (87.6)	1.00^
0-5+	31 (18.0)	37 (18.7)	0.96 (0.48-1.92)	22 (22.5)	25 (24.7)	1.10 (0.46-2.62)	9 (12.2)	12 (12.4)	1.16 (0.31-4.33)
Ham portions (each 50 g)/week									
0-0.5	103 (56.9)	98 (49.5)	1.00^	54 (52.4)	51 (50.5)	1.00^	49 (62.8)	47 (48.5)	1.00^
1	34 (18.8)	49 (24.7)	0.79 (0.40-1.55)	20 (19.4)	22 (21.8)	1.59 (0.53-4.82)	14 (18.0)	27 (27.8)	0.43 (0.16-1.12)
2+	44 (24.3)	51 (25.8)	0.94 (0.50-1.75)	29 (28.2)	28 (27.7)	1.33 (0.57-3.12)	15 (19.2)	23 (23.7)	0.52 (0.18-1.47)
P for trend			0.762			0.470			0.120
Sausages portions (each 50 g)/week									
0-0.5	119 (66.1)	130 (65.7)	1.00^	57 (55.9)	60 (59.4)	1.00^	62 (79.5)	70 (72.2)	1.00^
1	30 (16.7)	30 (15.1)	0.83 (0.39-1.74)	22 (21.6)	17 (16.8)	1.15 (0.41-3.24)	8 (10.3)	13 (13.4)	0.66 (0.19-2.27)
2+	31 (17.2)	38 (19.2)	0.86 (0.42-1.75)	23 (22.6)	24 (23.8)	0.94 (0.38-2.37)	8 (10.3)	14 (14.4)	0.92 (0.24-3.47)
P for trend			0.615			0.938			0.724
Milk cups (each 225 g)/week									
0-0.5	102 (56.4)	106 (53.5)	1.00^	55 (54.5)	54 (53.5)	1.00^	47 (58.8)	52 (53.6)	1.00^
1-6	19 (10.5)	32 (16.2)	0.68 (0.30-1.52)	6 (6.0)	22 (21.8)	0.33 (0.10-1.07)	13 (16.3)	10 (10.3)	1.98 (0.51-7.63)
7+	60 (33.1)	60 (30.3)	0.85 (0.46-1.55)	40 (39.6)	25 (24.8)	1.63 (0.64-4.18)	20 (25.0)	35 (36.1)	0.56 (0.23-1.39)
P for trend			0.537			0.538			0.252
Eggs number/week									

0-0.5	71 (38.6)	59 (29.8)	1.00^	42 (40.4)	32 (31.7)	1.00^	29 (36.3)	27 (27.8)	1.00^
1	48 (26.1)	55 (27.8)	0.99 (0.51-1.92)	26 (25.0)	22 (21.8)	1.51 (0.55-4.18)	22 (27.5)	33 (34.0)	0.77 (0.28-2.15)
2+	65 (35.3)	84 (42.4)	0.54 (0.29-1.00)	36 (34.6)	47 (46.5)	**0.38 (0.16-0.94)**	29 (36.3)	37 (38.1)	0.96 (0.37-2.47)
P for trend			**0.045**			**0.041**			0.962
Potatoes/week									
0-0.5	65 (35.9)	71 (35.9)	1.00^	35 (33.7)	30 (29.7)	1.00^	30 (39.0	41 (42.3)	1.00^
1	42 (23.2)	52 (26.2)	0.85 (0.42-1.69)	23 (22.1)	24 (23.8)	0.72 (0.25-2.10)	19 (24.7)	28 (28.9)	0.76 (0.28-2.12)
2+	74 (40.9)	75 (37.9)	1.31 (0.71-2.42)	46 (44.2)	47 (46.5)	0.80 (0.31-2.08)	28 (36.4)	28 (28.9)	2.26 (0.88-5.82)
P for trend			0.398			0.684			0.130
Carrots/week									
0-0.5	87 (47.8)	83 (41.9)	1.00^	53 (51.0)	45 (44.6)	1.00^	34 (43.6)	38 (39.2)	1.00^
1	48 (26.4)	50 (25.3)	0.79 (0.41-1.53)	29 (27.9)	23 (22.8)	1.03 (0.38-2.78)	19 (24.4)	27 (27.8)	0.53 (0.20-1.43)
2+	47 (25.8)	65 (32.8)	0.64 (0.35-1.18)	22 (21.1)	33 (32.7)	0.41 (0.16-1.03)	25 (32.1)	32 (33.0)	0.80 (0.31-2.05)
P for trend			0.153			0.072			0.592
Spinach portions (each 200 g)/week									
0-0.5	91 (50.6)	98 (49.8)	1.00^	54 (53.0)	54 (53.5)	1.00^	37 (47.4)	44 (45.8)	1.00^
1	54 (30.0)	66 (33.5)	1.35 (0.75-2.45)	30 (29.4)	27 (26.7)	1.96 (0.78-4.91)	24 (30.8)	39 (40.6)	1.14 (0.47-2.77)
2+	35 (19.4)	33 (16.7)	1.40 (0.69-2.87)	18 (17.6)	20 (19.8)	0.73 (0.27-2.01)	17 (21.8)	13 (13.5)	2.59 (0.82-8.13)
P for trend			0.272			0.897			0.140
Tomatoes portions (each 150 g)/week									
0-1	85 (47.0)	79 (39.9)	1.00^	49 (47.6)	41 (40.6)	1.00^	36 (46.2)	38 (39.2)	1.00^
2-3	61 (33.7)	78 (39.4)	0.88 (0.49-1.57)	35 (34.0)	40 (39.6)	0.60 (0.25-1.45)	26 (33.3)	38 (39.2)	0.96 (0.39-2.33)
4+	35 (19.3)	41 (20.7)	0.95 (0.48-1.89)	19 (18.5)	20 (19.8)	0.77 (0.26-2.28)	16 (20.5)	21 (21.7)	0.91 (0.31-2.66)
P for trend			0.824			0.521			0.862
Peppers/week									
0-0.5	135 (75.4)	133 (67.5)	1.00^	75 (72.8)	65 (65.0)	1.00^	60 (79.0)	68 (70.1)	1.00^
1	25 (14.0)	37 (18.8)	0.58 (0.28-1.19)	16 (15.5)	22 (22.0)	0.77 (0.29-2.06)	9 (11.8)	15 (15.5)	0.35 (0.10-1.18)
2+	19 (10.6)	27 (13.7)	0.74 (0.33-1.63)	12 (11.7)	13 (13.0)	0.86 (0.28-2.71)	7 (9.2)	14 (14.4)	0.74 (0.22-2.53)
P for trend			0.232			0.675			0.318
Salad portions (each 100 g)/week									
0-2	84 (46.1)	86 (43.5)	1.00^	55 (52.9)	48 (47.5)	1.00^	29 (37.2)	38 (39.2)	1.00^
3-4	44 (24.2)	48 (24.2)	0.83 (0.44-1.56)	27 (26.0)	22 (21.8)	1.09 (0.42-2.84)	17 (21.8)	26 (26.8)	0.45 (0.17-1.20)

5+	54 (29.7)	64 (32.3)	1.14 (0.60-2.14)	22 (21.1)	31 (30.7)	0.82 (0.31-2.16)	32 (41.0)	33 (34.0)	1.17 (0.46-3.01)
P for trend			0.742			0.707			0.792
Cabbage/week									
0-0.5	100 (55.9)	104 (52.8)	1.00^	58 (57.4)	55 (55.0)	1.00^	42 (53.9)	49 (50.5)	1.00^
1	27 (15.1)	39 (19.8)	0.75 (0.37-1.51)	16 (15.8)	18 (18.0)	1.10 (0.41-2.95)	11 (14.1)	21 (21.7)	0.57 (0.18-1.78)
2+	52 (29.0)	54 (27.4)	0.81 (0.44-1.52)	27 (26.7)	27 (27.0)	0.80 (0.31-2.08)	25 (32.1)	27 (27.8)	0.89 (0.36-2.21)
			0.473			0.702			0.764
Apples and pears/week									
0-2	104 (57.4)	115 (58.1)	1.00^	58 (56.3)	58 (57.4)	1.00^	46 (59.0)	57 (58.8)	1.00^
3-5	32 (17.7)	27 (13.6)	1.65 (0.78-3.51)	19 (18.5)	16 (15.8)	1.57 (0.57-4.37)	13 (16.7)	11 (11.3)	1.85 (0.53-6.51)
6+	45 (24.9)	56 (28.3)	0.71 (0.38-1.33)	26 (25.2)	27 (26.7)	0.77 (0.30-1.98)	19 (24.4)	29 29.9)	0.49 (0.19-1.27)
P for trend			0.425			0.752			0.193
Citrus cups/week [seasonally]									
0-0.5	76 (42.0)	71 (35.9)	1.00^	47 (45.6)	39 (38.6)	1.00^	29 (37.2)	32 (33.0)	1.00^
1-3	46 (25.4)	50 (25.2)	1.09 (0.56-2.13)	25 (24.3)	21 (20.8)	1.04 (0.35-3.13)	21 (26.9)	29 (29.9)	1.20 (0.47-3.10)
4+	59 (32.6)	77 (38.9)	0.86 (0.45-1.64)	31 (30.1)	41 (40.6)	0.50 (0.19-1.28)	28 (35.9)	36 (37.1)	1.74 (0.62-4.91)
P for trend			0.647			0.131			0.298
Melon portions (each 75 g)/week [seasonally]									
0-0.5	73 (40.3)	91 (46.0)	1.00^	49 (47.6)	54 (53.5)	1.00^	24 (30.8)	37 (38.1)	1.00^
1-2	65 (35.9)	57 (28.8)	1.80 (0.96-3.38)	31 (30.1)	24 (23.8)	2.44 (0.93-6.45)	34 (43.6)	33 (34.0)	1.58 (0.63-3.98)
3+	43 (23.8)	50 (25.2)	1.41 (0.72-2.75)	23 (22.3)	23 (22.8)	1.52 (0.56-4.10)	20 (25.6)	27 (27.8)	1.68 (0.60-4.66)
P for trend			0.261			0.298			0.310
Olive oil									
Not used/Scarce	29 (15.7)	30 (15.1)	1.00^	13 (12.5)	20 (19.8)	1.00^	16 (19.8)	10 (10.3)	1.00^
Normal use	136 (73.5)	137 (69.2)	0.98 (0.50-1.94)	78 (75.0)	66 (65.4)	2.32 (0.81-6.59)	58 (71.6)	71 (73.2)	0.45 (0.15-1.31)
High use	20 (10.8)	31 (15.7)	0.90 (0.36-2.26)	13 (12.5)	15 (14.9)	2.31 (0.59-9.09)	7 (8.6)	16 (16.5)	0.34 (0.08-1.40)
P for trend			0.837			0.199			0.122
Seed oil									
Not used	144 (79.1)	148 (75.5)	1.00^	85 (83.3)	73 (73.7)	1.00^	59 (73.8)	75 (77.3)	1.00^
Scarce	26 (14.3)	40 (20.4)	0.58 (0.29-1.15)	10 (9.8)	20 (20.2)	**0.30 (0.10-0.87)**	16 (20.0)	20 (20.6)	1.00 (0.36-2.81)
Normal use or High use	12 (6.6)	8 (4.1)	1.40 (0.42-4.69)	7 (6.9)	6 (6.1)	0.78 (0.15-3.98)	5 (6.3)	2 (2.0)	6.27 (0.56-69.9)

P for trend			0.588			0.120			0.278
Margarine									
Not used	174 (96.1)	188 (95.9)	1.00^	97 (96.0)	96 (97.0)	1.00^	77 (96.3)	92 (94.9)	1.00^
Scarce	3 (1.7)	6 (3.1)	1.35 (0.25-7.43)	2 (2-0)	2 (2.0)	2.15 (0.16-29.6)	1 (1.3)	4 (4.12)	0.67 (0.05-8.59)
Normal use or High use	4 (2.2)	2 (1.0)	1.40 (0.20-9.98)	2 (2.0)	1 (1.0)	1.48 (0.06-37.7)	2 (2.5)	1 (1.0)	2.31 (0.14-38.9)
P for trend			0.648			0.624			0.719
Water/per day									
≤1 liter	59 (48.4)	80 (47.3)	1.00^	26 (40.6)	37 (43.0)	1.00^	33 (56.9)	43 (51.8)	1.00^
>1 liter	63 (51.6)	89 (52.7)	0.84 (0.43-1.65)	20 (59.4)	49 (57.0)	0.62 (0.19-2.00)	25 (43.1	40 (48.2)	0.89 (0.36-2.23)

Significant estimates at a 0.05 level are in bold.

*Estimated using unconditional multiple logistic regression models after adjustment for sex, age, education, body mass index, smoking status, alcohol consumption, and hearing loss

^ Reference category.

°The sum does not add up to the total number of the subjects due to some missing values.
